# Elevated Vancomycin Trough Concentration: Increased Efficacy and/or Toxicity? 

**Published:** 2014

**Authors:** Sepideh Elyasi, Hossein Khalili, Simin Dashti-Khavidaki, Hamid Emadi-Koochak, Amirhooshang Mohammadpour, Alireza Abdollahi

**Affiliations:** a *Department of Clinical Pharmacy, Faculty of Pharmacy, Mashhad University of Medical Sciences, Mashhad, Iran.*; b *Department of Clinical Pharmacy, Faculty of Pharmacy, Tehran University of Medical Sciences, Tehran, Iran.*; c *Department of Infectious Diseases, Faculty of Medicine, Tehran University of Medical Sciences, Tehran, Iran.*; d *Department of Pathology, Faculty of Medicine, Tehran University of Medical Sciences, Tehran, Iran.*

**Keywords:** Vancomycin, Efficacy, Toxicity, Trough level

## Abstract

Vancomycin susceptibility of methicillin-resistant *Staphylococcus aureus* has been changed over time and its average minimum inhibitory concentration increased from 1.5 to 1.75 mg/L.A recently published guideline by the American Society of Health Pharmacist recommended a daily dose of 15-20 mg/Kg every 8 to 12 hours of vancomycin to achieve a trough concentration between 15-20 mg/L for treatment of severe infections.

Medical records of 69 patients from infectious ward of Imam Khomeini hospital, with suspected or confirmed gram-positive infection who had at least one trough level of vancomycin, were evaluated regarding vancomycin therapeutic goal; efficacy and renal safety. Most of patients (60.6%) with severe infections did not achieve the recommended vancomycin trough level during treatment course. Time to normalization of the signs and symptoms of infection did not correlate with the patients’ serum vancomycin trough levels. At the end of treatment course, there was no significant correlation between patients’ creatinine clearance and vancomycin trough levels (P=0.32). However, patients’cratinine clearance showed a negatively significant correlation with trough level of vancomycin (P=0.01). Vancomycin induced nephrotoxicity was detected in 4.3% of the patients. These data showed that vancomycin trough level may not necessarily assure treatment success, and also it would not essentially predict the risk of vancomycin induced nephrotoxicity. However, more well designed studies with larger sample size needed for better clinical and practical judgment.

## Introduction

Noscocomial infections due to gram-positive bacteria, especially methicillin-resistant *Staphylococcus aureus* (MRSA), is one of the main causes of morbidity and mortality in hospitalized patients. Since 1958, vancomycin, a glycopeptide antibiotic, has been accepted for treatment of Gram-positive infections, and it remains one of the first antibiotic choices for the treatment of severe MRSA infections (1-8). Inadequate vancomycin dosing is one of the major proposed causes of treatment failure in MRSA infected patients, despite confirmed *in-vitro* susceptibility. Vancomycin susceptibility of MRSA has been changed over time and its average minimum inhibitory concentration increased from 1.5 to 1.75 mg/L (2-3, 9). A recently published guideline by the American Society of Health Pharmacist (ASHP), and the Infectious Disease Society of America (IDSA), and the Society of Infectious Diseases Pharmacist (SIDP) recommended a daily dose of 15-20 mg/Kg every 8 to 12 hours (based on actual body weight) of vancomycin to maintain a trough concentration between 15-20 mg/L for treatment of severe infections such as bacteremia, endocarditis, osteomyelitis, meningitis and pneumonia (1-3, 9). However, some new studies indicated that the higher serum trough levels of vancomycin may not assure treatment success, at least in some types of infections (1,6,10).

Vancomycin induced nephrotoxicity (VIN) is an important consideration with the recommended high trough concentration of vancomycin (1-4, 9-11). VIN defined as a rise in serum creatinine by >50% during treatment with vancomycin, although it is not the best predictor of nephrotoxicity (11-13).

Several risk factors have been identified for VIN, which high trough vancomycin level (especially >20 mg/L) or doses (>4 g/day), concomitant use of nephrotoxic agents, prolonged therapy (more than 7 days), and admission to an intensive care unit (especially prolonged stay) are the most common ones (1-4, 14). Numerous antioxidants such as erdosteine, vitamin E, vitamin C, N-acetylcysteine, caffeic acid phenethyl ester, and erythropoietin proposed for preventing VIN (15). By the way, before using these agents in clinical practice, their efficacy should be evaluated in randomized controlled human studies. Several studies have reported a relationship between vancomycin serum trough concentrations and its renal toxicity, but actually, this causality has not been definitely established (1-4, 9, 10, 16-17). Also correlation between vancomycin serum trough concentration and its clinical efficacy is controversial (1-4). In this study correlation between vancomycin trough levels and its efficacy and/or renal toxicity have been evaluated. 

## Experimental

*Methods*

This prospective, observational study was performed during a 1.5 year period from October 2012 to March 2013 in the Infectious Diseases Ward of Imam Khomeini Hospital, main and referral teaching hospital affiliated to Tehran University of Medical Sciences, Tehran, Iran. The Institutional Review Board (IRB) and the Medical Ethics Committee of the hospital approved the study. During the study period, all patients admitted to infectious diseases ward of hospital and who received vancomycin (VanKo, JaberebneHayyan Pharmaceutical Company, Tehran, Iran) during their treatment course, were recruited. Patients with known risk factors for vancomycin induced nephrotoxicity such as baseline renal and/or hepatic insufficiency, diabetes mellitus and use of concomitant nephrotoxic agents were excluded. Finally sixty-nine patients with at least one trough vancomycinconcentration at steady state time completed the study.All patients signed consent form. 

The patients’ demographic data including sex, age, weigh, height, body mass index (BMI), initial temperature, RR (respiratory rate), heart rate (HR), blood pressure (BP), white blood cell count (WBC), serum creatinine (Scr) and also meanwhile the treatment course were recorded by a trained clinical pharmacist and creatinine clearance (Clcr) were calculated based on Cockroft-Gault formula.Vancomycin induced nephrotoxicity has been defined as a 0.5 mg/dL elevation in serum creatinine (2).

Moreover the patients’ type of infection and microbial culture results were identified and time course for resolution of fever, tachycardia, and leukocytosis were evaluated. Vancomycin treatment data including vancomycin dose regimen, total daily dose, dosing based on patient’s body weight and duration of therapy were collected. Patients with fever>38°C, elevated white blood cells, and persisting cultures following 5 days of vancomycin administration was considered as vancomycin treatment failure (1). The patients’ blood samples for measurement of trough vancomycin serum concentrations were collected at least after administering 4 doses of vancomycin to maintain steady state. The samples were drawn half an hour before infusion of next vancomycin dose and delivered to the laboratory in two hours. Vancomycin serum concentration was measured based of the defined radioimmunoassay method (ELISA Kits) (5). 

*Statistical analysis*

Statistical Package for the Social Sciences (SPSS) version 11.5 (SPSS Inc., USA) was used for descriptive statistical analysis. Mean values ± standard deviation were determined for all continuous variables. They were stratified by vancomycin trough concentrations (<5, 5-10 and >10 mg/L).Independent sample t-test was used for comparison of patients’ data in <3g/day and ≥3g/day vancomycin dose and One-Way ANOVA was used for comparison of data between three groups of patients based on serum vancomycin trough level. Also Pearson chi-square was used for evaluation of correlations.

## Results

Patients’ demographic data as whole and regarding different range of serum vancomycin trough levels were summarized in Table 1.

**Table 1:** Demographic data of the patients regarding their serum vancomycin trough concentrations.

**Sex (male) (%)**		**Age (years)**			**Weight (Kg)**	**BMI (Kg/m** ^2^ **)**	**Baseline serum creatinine (mg/dl)**	**Clcr (mL/min)**	**Baseline temperature (°C)**	**Daily total dose (mg)**	**Weight based dose (mg/Kg/d)**
All patients (69)											
79.4	50.15± 17.27		71.02 ± 11.05			24.89 ± 4.1	1.18 ± 0.78	78.78 ± 33.44	37.64 ± 0.74	1946 ± 879	33.73 ± 10.6
Patients with serum vancomycin trough concentrations < 5 mg/L (12)											
90.9	34.55 ± 19.76			66.5 ± 4.74		22.92 ± 1.24	1.6 ± 0.95	96.22 ± 33.47	37.84 ± 1.26	2204 ± 813	33.44 ± 11.94
Patients with serum vancomycin trough levels ranging from 5 to 10 mg/L (13)											
75		57.6 ± 19.1			72.27 ± 11.26	25.49 ± 6.81	1.42 ± 0.92	74.9 ± 36.52	37.45 ± 0.64	1777 ± 986	25.47 ± 15.84
Patients with serum vancomycin trough concentrations > 10 mg/L (44).											
78		51.53 ± 14.88			70.79 ± 11.37	25.01 ± 3.39	1.18 ± 0.83	76.63 ± 33.09	37.63 ± 0.64	2079 ± 854	32.78 ± 12.32
p-value	0.64	0.03*			0.63	0.52	0.4	0.19	0.35	0.92	0.85

The patients' demographic data was compared regarding their category of serum vancomycin trough level (<5, 5-10, and >10 mg/dl). One-way ANOVA was used for statistical comparison.

Serum vancomycin trough concentration > 10 mg/L, between 5-10 mg/L and less than 5 mg/L was respectively detected in 63.7%, 18.8% and 17.5% of the patients. There was no significant difference between demographic data of patients in the three categories of vancomycin trough level, except for age. Patients with serum vancomycin trough levels less than 5 mg/L were younger than other groups and had higher Clcr. So these patients cleared vancomycin more rapidly and consequently achieved less vancomycin serum level. 

**Figure 1:**
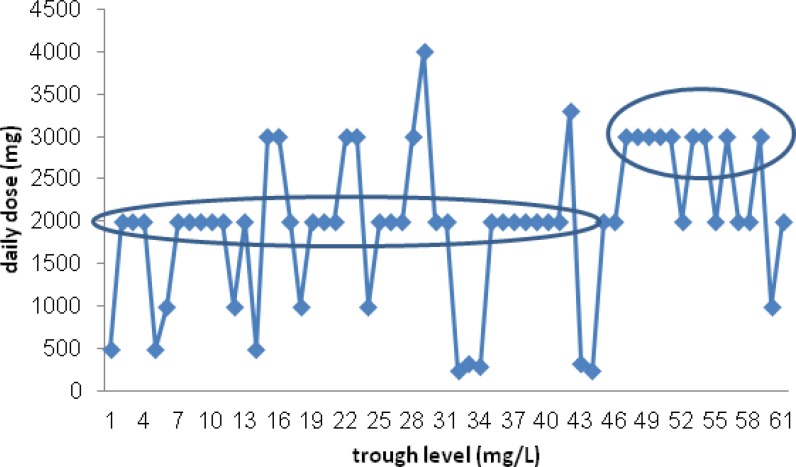
The patients’ vancomycin serum trough levels regarding received daily dose

The patients’ type of infection was categorized in Table 2. Soft tissue infections were common infection and were detected in about half of the patients. Thirty-three patients (47.8%) had at least one biological sample culture in their treatment course, which were positive in about half of them (16 patients). MRSA was the most common isolated microorganism (58.8%). Time (days) to negative culture was compared between three group and no significant difference was detected (P=0.22). There was no any correlation between the patients’ serum vancomycin trough levels and the type of infections (P = 0.21). Actually we could not find a significant correlation between vancomycin total daily dose (p = 0.74), per Kg daily dosing (p= 0.97) and vancomycin trough level. However most of patients receiving ≥3 g/day of vancomycin, had higher (but not significant) serum trough level than who received <3 g/day (P=0.15). (Figure 1)

**Table 2:** The patients’ types of infections

**Type of infection**	**Frequency (%)**
Soft tissue infections	45.9
Meningitis	31.1
Bacteremia	1.6
Pneumonia	18
Endocarditis	3.3

The patients’ types of infections were categorized based on the frequency.

The patients' vancomycin serum level (mg/L) plotted again weight based vancomycin daily dose (mg/Kg/d). There was not significant correlation between serum vancomycin trough levels and weight based daily dose of drug (mg/Kg/d)

Most patients (72.4%) with soft tissue infections achieved IDSA recommended serum trough level of vancomycin*i.e.* 10-15 mg/L (5). For other infections including meningitis, endocarditis, pneumonia and bacteremia the recommended serum trough level is 15-20 mg/L and only 39.4% of the patient achieved this goal. In 51% of patients vancomycin dosing was compatible with kullar *et al. *(21) nomogram and 64% of them achieved the recommended therapeutic serum trough levels. 

**Figure 2:**
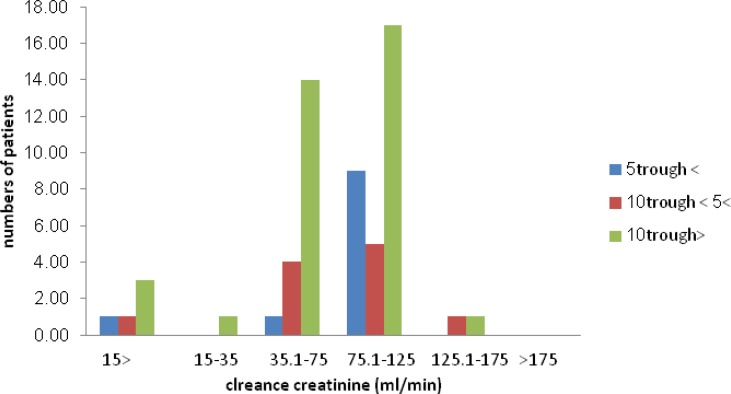
The patients’ creatinine clearance status regarding their serum vamcomycin trough levels.

The patients’ creatinine clearance status (based on the Cockroft-Gault formula) was plotted regarding serum vamcomycin trough levels. No significant correlation was found between these parameters. 

Patients with serum vancomycin trough levels <5 mg/L received shorter course of vancomycin therapy compared with other groups of patients (P = 0.02). Also most of these patients received vancomycin with total daily dose of less than 3 g/day. 

Times (days) to a normal WBC count, hemodynamic parameters (BP and HR), and becoming afebrile, as clinical criteria for evaluation of response to therapy, were compared between the patients regarding their serum vancomycin trough levels which was not significantly different (P= 0.62, 0.32 and 0.87, respectively). In other hand, days last for normalization of temperature and HR was significantly shorter in patients who receive higher daily dose of vancomycin (≥3g/day) (P=0.04 and 0.005 respectively) but was not significant for resolution of patients’ leukocytosis (P=0.61) (Table 3).Tobacco use and diabetes mellitus were similarly distributed in both groups (𝓧^2^=20, P=0.5and 𝓧^2^=27.9, P= 0.34), but cardiovascular diseases were more common in patients who receive <3g/day vancomycin, which could affect heart rate of patients (P=0.02).At the end of treatment course, there was no significant difference between the patients’ Clcr regarding their vancomycin serum trough levels (P=0.32) but surprisingly Clcr was significantly higher in the patients who received higher dose of vancomycin (≥3g/day) (P<0.001), despite of their insignificant difference in baseline Scr (P=0.37).

**Table 3:** Time to normalization of the signs and symptoms of infection

**Parameter**	**Trough level <5 mg/L**	**5≤ Trough level ≤10 mg/L**	**Trough level >10 mg/L**
WBC count normalization time (day)	4.14 ± 4.49	4.67 ± 4.23	2.85 ± 4.56
HR normalization time (day)	4.29 ± 3.6	5.17 ± 3.31	3.16 ± 2.59
Time to become afebrile (day)	2 ± 2.65	1.62 ± 2.83	2.17 ± 2.39

**Figure 3:**
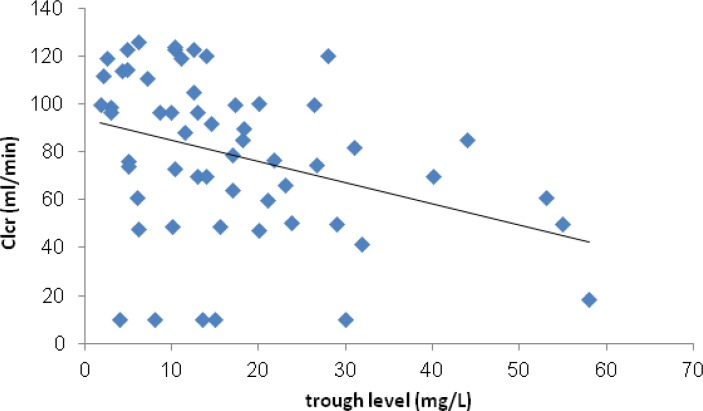
Correlation between Clcr and serum trough level of vancomycin

However Clcr negatively correlated with serum trough level of vancomycin (P=0.01). (Figure 3) Just 3 (4.7%) of patients experienced VIN. In these patients vancomycin serum trough levels were >10 mg/L and Scr normalized following discontinuation or decrement of vancomycin dose. Based on the definition of treatment failure, 17 (24.6%) of patients did not response to therapy. Sixteen (94.1%) of these patients received vancomycin daily dose of <3 g. 

Correlation between Clcr and serum trough level of vancomycin was plotted. A non-significant negative correlation between these parameters has been shown.

## Discussion

Up to recent years vancomycin usually is prescribed as a consistent dose (1 g every 12 h) for all adult hospitalized patients and dose adjustment just performed on the basis of Clcr not body weight or serum trough level. After availability and routine vancomycin therapeutic drug monitoring, initial weight-based dosing and following adjustment based on serum trough level is recommended by a recent consensus guideline (1). MRSA infections are the main cause of hospital morbidity and mortality (2-8). Following several reports regarding decreased MRSA susceptibility to vancomycin, higher serum trough level was recommended for treatment of severe nosocomial infections such as bacterimia, meningitis, pneumonia and endocarditis (1). For archiving this goal of therapy, most patients with normal renal function needed higher and weight based vancomycin doses than usual practice. For patients with soft tissue infections, which recommended trough level is lower (10-15 mg/L), most of patients achieved the goal levels, but in serious infections like meningitis that new IDSA guideline recommended higher trough level (15-20 mg/L), less than half of patients acquired this goal. Our findings show that conventional practice for vancomycin dosing is not rational (18-20). Recent population based nomograms was designed and recommended for initial dosing of vancomycin (18). Unfortunately these data are not available for Iranian population. Population pharmacokinetic studies regarding vancomycin dosing in Iranian population warranted to develop appropriate nomograms for vancomycin dosing in our hospitals. As suspected, higher doses of vancomycin should attain higher serum trough levels (5), but we could not find a significant correlation between vancomycin dose and consequent serum trough level, although in most of patients who received <3 g/day vancomycin, lower serum levels was detected in comparison with patients who received ≥ 3 g/day. Changes in patient's physiological parameters including temperature, HR, RR, BP following infections may affect vancomycin clearance and accordingly serum trough levels. For considering these probable confounding factors, future studies must be focused on evaluation of infection type effects on pharmacokinetic behavior of vancomycin. Patients who received higher daily doses of vancomycin showed more rapid resolution of predictive signs and symptoms of infection such as leukocytosis, tachycardia, and fever but we could not find a significant correlation between serum vancomycin trough level and these parameters. Some of the past in-vitro and *in-vivo* studies also reported the same results (1, 6, 10, 21).Most of included patients received vancomycin for more than 7 days. Eleven patients had Scr>1.2 g/dL at baseline, but just 3 of these patients experienced VIN. Incidence of VIN was reported as 5-35% in previous studies (2, 14, 22). In our study VIN was detected in 4.3% of patients that is compatible with previous results. Baseline patient's higher Scr defined as a predisposing factor for VIN in previous studies (1, 2, 4, 9). As VIN was detected in limited numbers of patients, we could not evaluate association between serum trough level of drug and incidence of VIN, although patients with VIN had serum trough level >10 mg/L. Most of the preceding data showed this correlation (1-4, 10, 9, 16-17).A non-significant correlation between duration of vancomycin therapy and occurrence of VIN was detected in our patients. Significant association between vancomycin duration of therapy and VIN was reported in previous studies (1-4).Based on the definition of treatment failure, 24.6% of patients experienced failure. Inadequate of vancomycin dosing is the main cause of the treatment failure that was detected in 94.1% of patients. 

This study has several limitations including observational type, small sample size and included various types of infections. Interventional studies with adequate sample size in each type of severe infections such as sepsis, endocarditis, meningitis and pneumonia should be designed in the future. There are limited data regarding vancomycin pharmacokinetic parameters in Iranian population. Multicenter studies needed to organize these data and to propose the best pharmacokinetic model and nomogram in hospitalized patients. For detection of VIN more sensitive and reliable biomarkers such as cystatin C, kidney injury molecule 1(KIM-1) and neutrophil gelatinase–associated lipocalin(NGAL) other than Scr must be considered. 
